# A fixed 20:1 combination of cafedrine/theodrenaline increases cytosolic Ca^2+^ concentration in human tracheal epithelial cells via ryanodine receptor-mediated Ca^2+^ release

**DOI:** 10.1038/s41598-023-43342-0

**Published:** 2023-09-27

**Authors:** Götz Schmidt, Gerrit Rienas, Sabrina Müller, Katrin Richter, Michael Sander, Christian Koch, Michael Henrich

**Affiliations:** 1 Department of Anesthesiology, Operative Intensive Care Medicine and Pain Therapy, Justus Liebig University of Giessen, Rudolf-Buchheim-Strasse 7, 35392 Giessen, Germany; 2 Department of General and Thoracic Surgery, Justus Liebig University of Giessen, Rudolf-Buchheim-Strasse 7, 35392 Giessen, Germany; 3 Department of Anesthesiology, Intensive Care Medicine, Emergency Medicine, Vidia St. Vincentius-Clinic Karlsruhe gAG, Karlsruhe, Germany

**Keywords:** Drug regulation, Calcium and vitamin D, Comorbidities

## Abstract

Mucociliary clearance is a pivotal physiological mechanism that protects the lung by cleaning the airways from pollution and colonization, thereby preventing infection. Ciliary function is influenced by various signal transduction cascades, and Ca^2+^ represents a key second messenger. A fixed 20:1 combination of cafedrine and theodrenaline has been widely used to treat perioperative hypotension and emergency hypotensive states since the 1960s; however, its effect on the intracellular Ca^2+^ concentration ([Ca^2+^]_i_) of respiratory epithelium remains unknown. Therefore, human tracheal epithelial cells were exposed to the clinically applied 20:1 mixture of cafedrine/theodrenaline and the individual substances separately. [Ca^2+^]_i_ was assessed by FURA-2 340/380 fluorescence ratio. Pharmacological inhibitors were applied to elucidate relevant signal transduction cascades, and reverse transcription polymerase chain reaction (RT-PCR) was performed on murine tracheal epithelium to analyze ryanodine receptor (RyR) subtype expression. All three pharmacological preparations instantaneously induced a steep increase in [Ca^2+^]_i_ that quickly returned to its baseline value despite the persistence of each substance. Peak [Ca^2+^]_i_ following the administration of 20:1 cafedrine/theodrenaline, cafedrine alone, and theodrenaline alone increased in a dose-dependent manner, with median effective concentrations of 0.35 mM (7.32 mM cafedrine and 0.35 mM theodrenaline), 3.14 mM, and 3.45 mM, respectively. When extracellular Ca^2+^ influx was inhibited using a Ca^2+^-free buffer solution, the peak [Ca^2+^]_i_ following the administration of cafedrine alone and theodrenaline alone were reduced but not abolished. No alteration in [Ca^2+^]_i_ compared with baseline [Ca^2+^]_i_ was observed during β-adrenergic receptor inhibition. Depletion of caffeine-sensitive stores and inhibition of RyR, but not IP_3_ receptors, completely abolished any increase in [Ca^2+^]_i_. However, [Ca^2+^]_i_ still increased following the depletion of mitochondrial Ca^2+^ stores using 2,4-dinitrophenol. RT-PCR revealed RyR-2 and RyR-3 expression on murine tracheal epithelium. Although our experiments showed that cafedrine/theodrenaline, cafedrine alone, or theodrenaline alone release Ca^2+^ from intracellular stores through mechanisms that are exclusively triggered by β-adrenergic receptor stimulation, which most probably lead to RyR activation, clinical plasma concentrations are considerably lower than those used in our experiments to elicit an increase in [Ca^2+^]_i_; therefore, further studies are needed to evaluate the ability of cafedrine/theodrenaline to alter mucociliary clearance in clinical practice.

## Introduction

Mucociliary clearance of the lower airways is a pivotal physiological mechanism that protects the lung by clearing the airways of pollution and colonization by pathogens, thereby preventing infection. Together with basal, suprabasal, and goblet cells, multiciliated epithelial cells constitute the complex mucociliary clearance function of the respiratory tract^1^. Ciliary cells prevent the accumulation of debris and colonization by microbial pathogens via an outward-directed transportation processes^1,2^. Cilia are hair-like protrusions located on the apical side of the respiratory epithelium along the airways; they are composed of unique structural proteins, including motor proteins, like the ATP-dependent dynein^2,3^. Due to their continuous synchronized and orally directed motion, inhaled particles and complex compositions of mucus, electrolytes, and endogenous defensive substances are removed from the lower airways and are subsequently coughed up under normal physiological conditions^1,4^. While the ciliary apparatus continuously beats without external stimulation, various events can provoke an increase in ciliary beating frequency, when necessary^1,4^. Exogenous, endogenous, and paracrine effects moderate ciliary activity, and Ca^2+^ functions as a central second messenger. Therefore, intracellular Ca^2+^ concentration ([Ca^2+^]_i_) represents a keystone regulating ciliary activity because most signal transduction cascades, which contribute to the ciliary beating frequency, induce at least a temporary increase in [Ca^2+^]_i_^1,4,5^. Intracellular Ca^2+^ is foremost stored in the endoplasmic reticulum (ER) and is released following inositol trisphosphate (IP_3_) or ryanodine receptor (RyR) activation^3^. Furthermore, the ER regulates [Ca^2+^]_i_ via the sarcoplasmic/endoplasmic reticulum calcium ATPase (SERCA), which pumps cytosolic Ca^2+^ ions to its internal stores, maintaining the balance of [Ca^2+^]_i_^6,7^. Mitochondria also contribute to intracellular Ca^2+^ homeostasis by buffering Ca^2+^ ions when [Ca^2+^]_i_ exceeds a threshold of 500 nM^8,9^. In addition, other cell organelles contribute to intracellular Ca^2+^ homeostasis, such as lysosomes, which contain Ca^2+^ but can also regulate mitochondrial Ca^2+^ dynamics, and peroxisomes; however, their individual influence on [Ca^2+^]_i_ in ciliary cells and mucociliary clearance remains unknown^6,10,11^. [Ca^2+^]_i_ is not only increased by Ca^2+^ release from intracellular stores; extracellular Ca^2+^ can enter the cell via plasma membrane-bound Ca^2+^ channels. Although voltage-gated calcium channels and transient receptor potential channels are located at the plasma membrane together, the predominant method of extracellular Ca^2+^ entry into non-excitable cells occurs via store-operated Ca^2+^ entry (SOCE)^12,13^. SOCE is triggered by an increase in [Ca^2+^]_i_ that is predominantly caused by the liberation of Ca^2+^ from internal stores^12^. Stromal interaction molecule proteins are located at the ER membrane, and they activate SOCE when Ca^2+^ is released^12,13^. Therefore, changes in [Ca^2+^]_i_ produce fundamental metabolic and functional alterations in ciliary cells and can be influenced by different drugs administered in various settings.

In Germany, intraoperative hypotension and emergency hypotensive states have been treated with a combination of cafedrine and theodrenaline (Akrinor®, Ratiopharm GmbH, Ulm, Germany) since the 1960s^14–16^. Covalently linked theophylline and norephedrine compose cafedrine, and theodrenaline is formed from theophylline and noradrenaline in the same manner^14,17^. Both compounds are administered in a fixed 20:1 combination of cafedrine and theodrenaline as an intravenous bolus in adults, and they restore mean arterial blood pressure by increasing preload, cardiac stroke volume, and cardiac output^14,15,18^. The clinical effects of cafedrine/theodrenaline are mediated through β_1_-adrenoreceptor and α-adrenoreceptor stimulation, and nonspecific inhibition of phosphodiesterases (PDEs) is believed to enhance their response^14,17^. In contrast with synthetic vasopressors (e.g., ephedrine, phenylephrine), systemic vascular resistance and heart rate remain mostly unaffected, which makes cafedrine/theodrenaline especially appealing in obstetric surgery^14,16,19^. Although cafedrine/theodrenaline has been widely used for decades, little is known about its pharmacodynamics or pharmacokinetics in specific end organs^14,16^. This is surprising as the unique combination of three single drugs (theophylline, norephedrine, noradrenaline) in a 20:1 mixture might produce different effects in vivo as recently shown in human atrial myocardium and internal mammary arteries^17^. However, the influence of cafedrine/theodrenaline on the [Ca^2+^]_i_ of human respiratory cilia cells has not yet been investigated. The aim of this study was to evaluate the influence of cafedrine/theodrenaline on the [Ca^2+^]_i_ of human tracheal epithelial cells. Cells were exposed to the clinically used 20:1 mixture of cafedrine/theodrenaline and the individual substances alone. Our experiments elucidated the effects of the individual components and identified the relevant pharmacological signaling cascades leading to alterations in [Ca^2+^]_i_. Therefore, we applied specific inhibitory substances inhibiting distinct signal transduction cascades, and we evaluated the origin of the released Ca^2+^. Furthermore, we used reverse transcription polymerase chain reaction (RT-PCR) to detect the messenger ribonucleic acid (mRNA) expression of key receptor subtypes influencing [Ca^2+^]_i_ following the administration of cafedrine/theodrenaline, cafedrine alone, and theodrenaline alone.

## Methods

### Drugs and buffer solutions

The historically established 20:1 ratio of cafedrine/theodrenaline refers to mass, not molarity. Therefore, due to the different molar masses of cafedrine (357.41 g/mol) and theodrenaline (375.38 g/mol), a 1 M solution of cafedrine/theodrenaline 20:1 with a molar mass of 7,882.98 g/mol consists of 1 M theodrenaline and approximately 21 M of cafedrine. Experiments were performed in HEPES solution consisting of 10 mM HEPES, 2.6 mM KCl, 2.5 mM CaCl_2_, 10 mM glucose, 125 mM NaCl, 1.2 mM KH_2_PO_4_, and 1.2 mM MgSO_4_. NaOH was used to adjust the pH to 7.4 at 30 °C. To realize experiments in Ca^2+^-free solutions, CaCl_2_ was substituted with 1 mM ethylene glycol tetraacetic acid. The following drugs were applied during the experiments: 2-aminoethoxydiphenylborane (2-APB, 40 µM diluted in 4 µl dimethyl sulfoxide [DMSO], TOCRIS Bioscience, Bristol, UK), 2,4-dinitrophenol (DNP, 25 µM diluted in 10 µl DMSO, Sigma-Aldrich, St. Louis, USA), cafedrine (3.14 mM diluted in 50 µl H_2_O, Arevipharma, Radebeul, Germany), cafedrine/theodrenaline 20:1 (0.38 mM diluted in 50 µl H_2_O, Akrinor®, Ratiopharm, Ulm, Germany), caffeine (30 mM, Roth, Karlsruhe, Germany), FURA-2 AM (2.5 µM diluted in 5 µl H_2_O, Biotium, Fremont, USA), ICI-118,551 (100 µM diluted in 10 µl H_2_O, TOCRIS Bioscience, Bristol, UK), KCl (200 mM diluted in 66.6 µl H_2_O), ryanodine (40 µM diluted in 16 µl DMSO, TOCRIS Bioscience, Bristol, UK), and theodrenaline (3.45 mM diluted in 50 µl H_2_O, Arevipharma, Radebeul, Germany). The stated drug concentrations were achieved during the experiments after applying the stock solution to the buffer solution in the recording chamber. In control experiments, the buffer solution or the solvent alone was applied to rule out any contribution of the buffer, the solvent, or the mechanical application process to the Ca^2+^ signals.

### Calcium imaging in isolated human tracheal epithelial cells

Human tracheal epithelial cells (HTEpC, C12644, PromoCell, Heidelberg, Germany) were cultured with the Airway Epithelial Growth Medium Kit (C-21160) containing the Airway Epithelial Growth Medium Supplement Pack (C-39160, both PromoCell, Heidelberg, Germany) in a humidified chamber at 37 °C with air containing 5% CO_2_. Cells from passage one to three were seeded onto laminin-coated coverslips for [Ca^2+^]_i_ measurements in 4-(2-hydroxyethyl)-1-piperazineethanesulfonic acid (HEPES) buffer. Dye loading was performed in the dark with 2.5 µM FURA-2 AM for 45 min at 37 °C. The cell-containing coverslip was then rinsed in fresh HEPES buffer and subsequently transferred to the recording chamber of an upright fluorescence microscope equipped with a 20 × immersion lens (BX50 WI, Olympus, Hamburg, Germany), where the coverslip was placed into a Delta T culture dish (Bioptechs, Butler, USA) containing 2 ml of fresh HEPES buffer. Excitation light was provided by a 50 W xenon lamp, and the microscope was equipped with a dichroic excitation longpass mirror at 400 nm. FURA-2 AM was excited at 340 nm and 380 nm while equipped with bandpass excitation filters. The emitted fluorescence was directed through a dichroic shortpass filter of 560 nm to a bandpass filter of 510 nm and was recorded with a scientific camera (SMX-150, Sumix, Oceanside, USA). Measurements of the FURA-2 AM 340/380 ratio were performed every second for 1,000 s using an automated protocol of the TiLLvisION Imaging software program (Till Photonics, Gräfeling, Germany). Cafedrine, theodrenaline, or 20:1 cafedrine/theodrenaline were added following a 100-s resting period to ensure adequate baseline calibration. After 800 s, 200 mM potassium chloride (KCl) was added to the buffer solution to completely invert the membrane potential, leading to massive influx of Ca^2+^ indicating the integrity of the analyzed cells up to the end of the respective experiment.

### RNA extraction from murine tissues

Male C57BL6J mice (n = 5) weighing 25–35 g (aged 12–15 weeks) were purchased from Charles River (Sulzfeld, Germany) to characterize the expression of RyR in the respiratory epithelium. All procedures involving animals were conducted in compliance with the European legislation for the protection of animals used for scientific purposes, the ARRIVE guidelines and the standards for animal experiments according to the German animal welfare law and were approved by the local committee for animal care of the regional council of Giessen, Germany (Permit number 813_M, Regional Council of Giessen, Germany). After deep narcosis using 5% isoflurane (Baxter, Unterschleissheim, Germany), animals were sacrificed by cervical dislocation. Tracheae, tracheal epithelium, diaphragm, cardiac muscle, and skeletal muscle were subsequently collected. To obtain isolated tracheal epithelium, the epithelial layer was gently scrubbed from the opened trachea using a hygienic swab. All tissues were stored in RNA stabilization lotion (Invitrogen™ RNAlater™, Thermo Fisher Scientific, Waltham, USA) at − 20 °C until further processing. Tissue probes were then lysed in 350 µl RNeasy Lysis Buffer (Qiagen, Hilden, Germany) containing 3.5 µl ß-mercaptoethanol and they were subsequently homogenized in a tissue homogenizer (Precellys Evolution homogenizer, Bertin Technologies, Montigny-le-Bretonneux, France). RNA was extracted using the RNeasy Micro Kit, and DNA removal was performed using the RNase-Free DNase set (both Qiagen, Hilden, Germany).

### RT-PCR

cDNA synthesis was performed using the QuantiTect Reverse Transcription Kit (Qiagen, Hilden, Germany) according to the manufacturer’s protocol and stored at − 20 °C until further use. RyR-1, RyR-2, and RyR*-*3 expression in whole trachea and in the respiratory epithelium alone (each n = 5) was analyzed using specific primers at temperatures shown in Table 1. Primers were selected using the NCBI Primer designing tool (https://www.ncbi.nlm.nih.gov/tools/primer-blast, National Institutes of Health, Bethesda, MD, USA). Skeletal muscle (RyR-1), cardiac muscle (RyR-2), and diaphragm (RyR-3) served as positive controls, and H_2_O was used as negative control. Primer concentration was set at 0.2 µM and reactions were performed in a Mastercycler gradient (Eppendorf, Hamburg, Germany). TAQ polymerase (Qiagen, Hilden, Germany) was activated at 95° for 3 min, followed by 40 cycles consisting of a 1-min denaturation step at 95 °C, a 45-s annealing step at a primer-specific temperature (Table 1), and a 3-min extension step at 72 °C. PCR products were visualized using GelRed (Biotium, Fremont, CA, USA) with a digital imaging system (Vilber Lourmat, Eberhardzell, Germany) in agarose gel (1% TAE buffer) after electrophoresis at 100 V for 80 min. GeneRuler 100 bp (Thermo Fisher Scientific, Waltham, USA) was used to verify the proper size of the PCR products.

**Table 1:** List of murine ryanodine receptor (RyR) primers used for RT-PCR and their specific annealing temperatures.

Target	Gene	Sequence	Product length (bp)	Annealing temperature (°C)
RyR 1	*Ryr1*	Forward: 5’-CGCTCCCACTTCATCCCTAC-3’	385	55
		Reverse: 5’-CTCCTGCCTTGGCCATTTTG-3’		
RyR 2	*Ryr2*	Forward: 5’-ACCTACTCCGAAGGCTGGTGTT-3’	148	55
		Reverse: 5’-TTCTTCCGAGGCAGCACCAAAG-3’		
RyR 3	*Ryr3*	Forward: 5’-GACAGGACCAGGAACGGAAG-3’	315	58
		Reverse: 5’-GCTCCACCGTCTTTTCTGGA-3’		

### Statistical analysis

FURA-2 340/380 ratio was recorded from at least 30 cells per experimental setup. These 30 cells were collected from at least three different coverslips each evaluating 10 cells taken from cell passages one to three. Measurements were only included in the statistical analyses when clear response of the FURA-2-fluorescence ratio (doubling of the ratio) was detected after the application of KCl at the end of the experiments. Median effective concentrations (EC_50_) were calculated using the Hill equation. The Mann–Whitney U test was used to compare the peak measurement points from different experiments, while the Wilcoxon rank sum test was used to compare paired variables. In general, two-tailed values of *p* < 0.05 were considered statistically significant, while multiple comparison problems were counteracted by adjusting the α-level according to the Bonferrroni correction. GraphPad PRISM (Version 9.5.0, GraphPad Software, La Jolla, CA, USA) was used for statistical analysis and figure creation.

### Ethical approval

Permit number 813_M, Regional Council of Giessen, Germany.

## Results

### 20:1 cafedrine/theodrenaline, cafedrine, and theodrenaline induce a transient rise in [Ca^2+^]_i_

**Figure 1:**
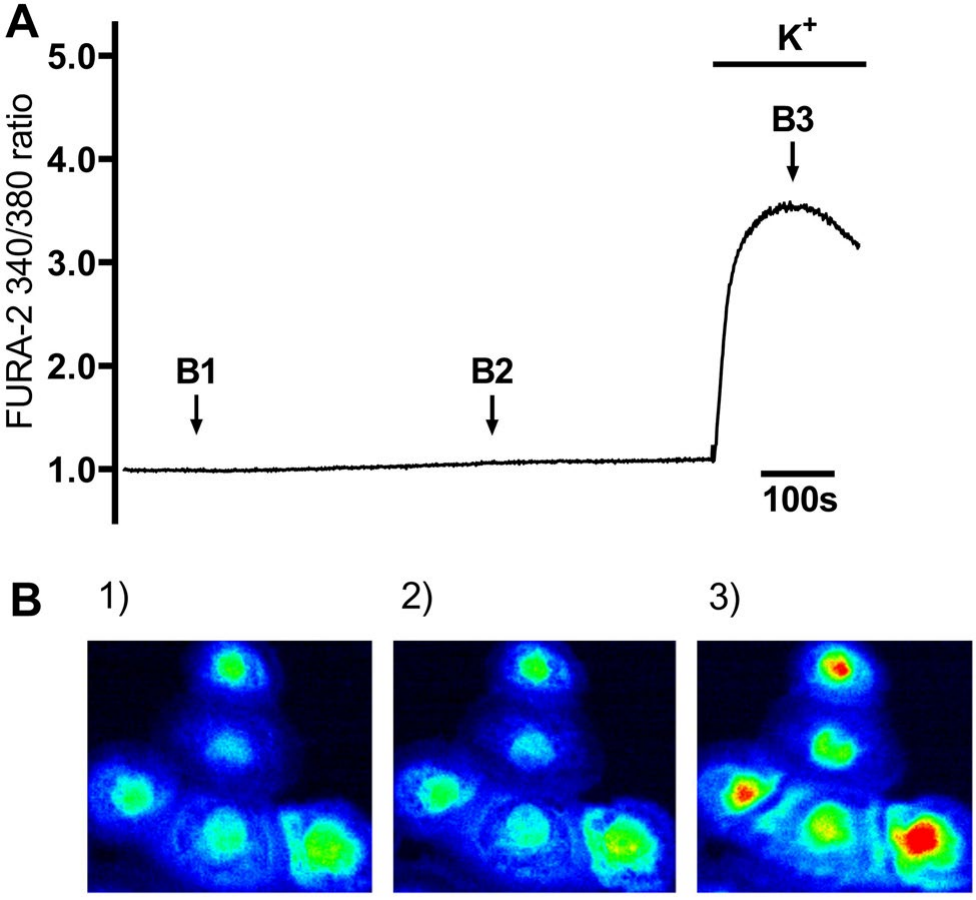
Intracellular Ca^2+^ measurements were performed on Human tracheal epithelial cells (HTEpC). Intracellular Ca^2+^ concentrations ([Ca^2+^]_i_) were recorded as Fura-2/AM (Fura-2) fluorescence intensity ratio of 340:380 nm excitation. (**A**) When FURA-2 340/380 ratio was normalized after a 100-s resting period, fluorescence ratio remained constant under resting conditions during the 800-s observation period, demonstrating no changes in [Ca^2^]_i_. Arrows indicate different timepoints of the FURA-2 340/380 ratio, which are illustrated in (**B1**–**B3**) after conversion into false colors. Potassium chloride (K^+^, 200 mM) completely inverted the membrane potential, leading to a strong increase in FURA-2 340/380 fluorescence of human tracheal epithelial cells.

**Figure 2:**
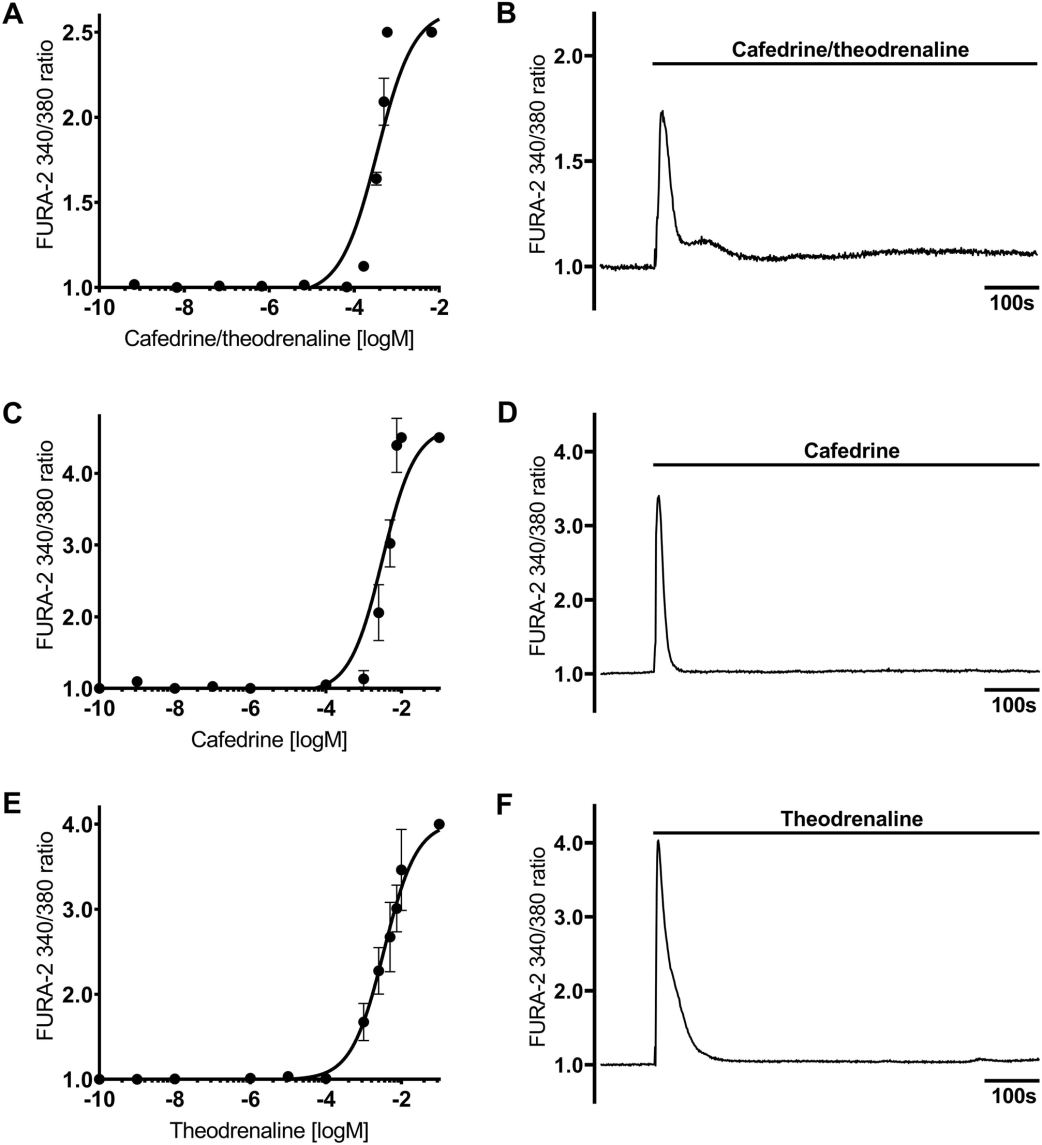
[Ca^2^]_i_ is increased by cafedrine/theodrenaline, cafedrine alone, and theodrenaline alone. Dose–response relationships of (**A**) 20:1 cafedrine/theodrenaline, (**C**) cafedrine alone, and € theodrenaline are described by the Hill equation. (**B**) Cafedrine/theodrenaline (0.38 mM), (**D**) cafedrine alone (3.14 mM), and (**F**) theodrenaline alone (3.45 mM) induced a steep increase in [Ca^2^]_i_; however, [Ca^2^]_i_ rapidly returned to its baseline value despite the continued presence of the applied substances. Application of potassium chloride (200 mM) confirmed the vitality of the included cells (not shown). FURA-2 340/380 ratio was normalized after a 100-s resting period, and each group consists of 30 cells from at least three different coverslips, ensuring independent measurements. Scale bar width represents 100 s. ⊥ SEM.

Under resting conditions, FURA-2 340/380 ratio remained constant during the whole observation period (FURA-2 340/380 ratio: 0.98 ± 0.02, Fig. 1). [Ca^2+^]_i_, indicated by FURA-2 340/380 ratio, sharply increased when KCl was applied at the end of the experiments, indicating the vitality of the human tracheal epithelial cells. This is demonstrated for all the experiments by an example in Fig. 1. Application of 20:1 cafedrine/theodrenaline, cafedrine alone, and theodrenaline increased [Ca^2+^]_i_ in a dose-dependent manner following the Hill equation (Fig. 2A,C,E). EC_50_ calculated for 20:1 cafedrine/theodrenaline, cafedrine, and theodrenaline were 0.35 mM (2.75 mg/ml consisting of 2.62 mg/ml [7.32 mM] cafedrine and 0.13 mg/ml [0.35 mM] theodrenaline), 3.14 mM (1.12 mg/ml), and 3.45 mM (1.30 mg/ml), respectively. All three substances provoked a sharp increase in [Ca^2+^]_i_ that was observed immediately, within 5 s after application (FURA-2 340/380 ratios of cafedrine/theodrenaline: 1.76 ± 0.06, cafedrine: 4.06 ± 0.24, theodrenaline: 4.38 ± 0.31; each *p* < 0.001; Fig. 2B,D,F). [Ca^2+^]_i_ subsequently returned to its baseline value despite the ongoing presence of the applied substances, and the steepest decrease was observed after the administration of cafedrine (Fig. 2D). The following experiments aimed to evaluate the proportion of extracellular Ca^2+^ entry contributing to the transient rise in [Ca^2+^]_i_.

### The increase in [Ca^2+^]_i_ depends on extracellular Ca^2+^ entry

**Figure 3:**
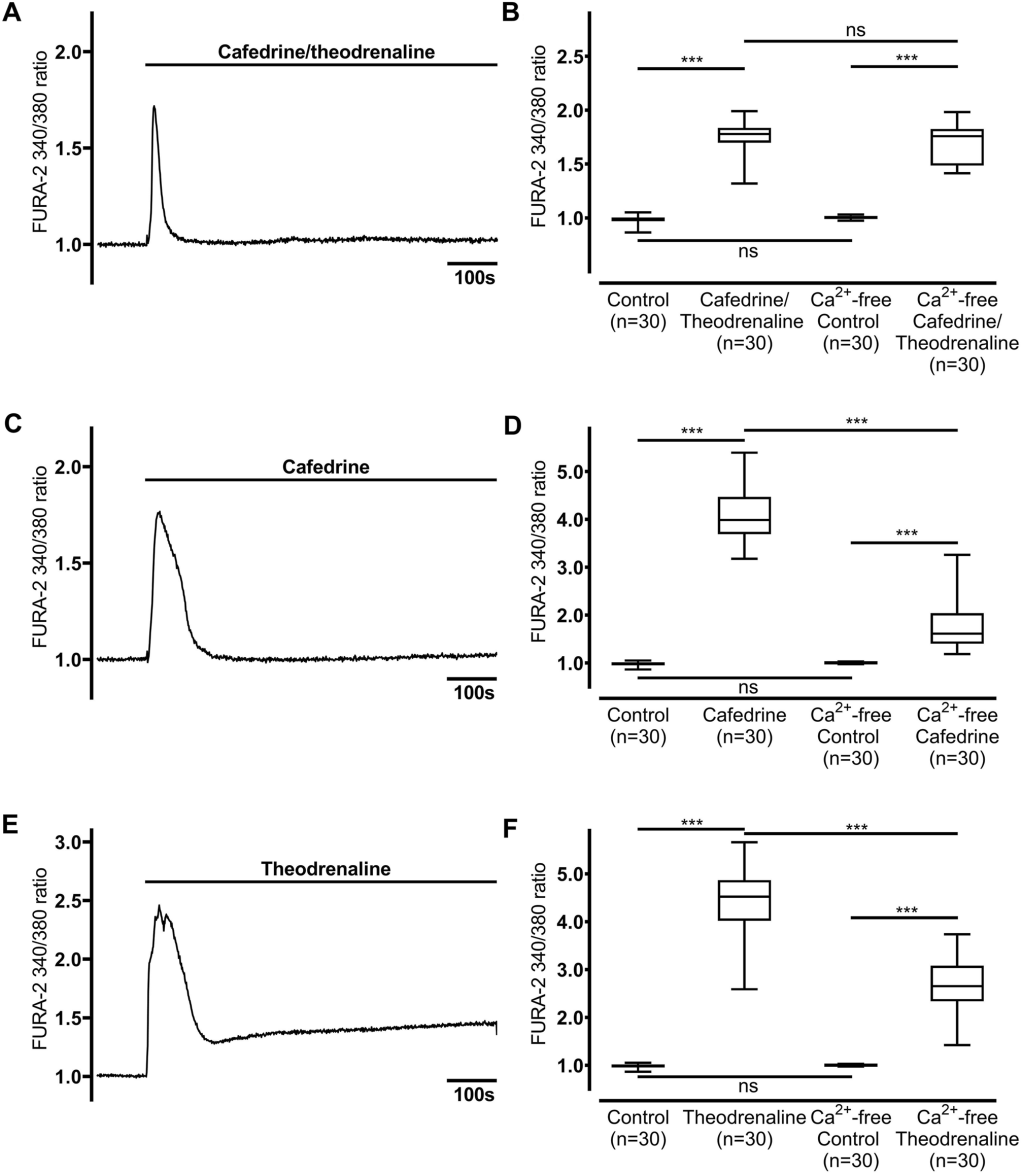
Increase in [Ca^2^]_i_ depends on extracellular Ca^2+^ influx following the application of cafedrine alone or theodrenaline alone. When experiments were performed in Ca^2^-free buffer, the maximum [Ca^2^]_i_ provoked by (**A, B**) 20:1 cafedrine/theodrenaline was comparable to the peak [Ca^2^]_i_ seen in Ca^2+^-containing buffer; however, peak [Ca^2^]_i_ observed after the application of (**C, D**) cafedrine alone and (**E, F**) theodrenaline alone was significantly reduced. When theodrenaline was applied, [Ca^2^]_i_ did not return to its baseline value during the whole observation period. Subsequent application of potassium chloride (200 mM) confirmed the vitality of the included cells (not shown). The control group is represented by the same 30 cells in each chart. FURA-2 340/380 ratio was normalized after a 100-s resting period, and each group consists of 30 cells from at least three different coverslips, ensuring independent measurements. Scale bar width represents 100 s. n = number of individual cells, ***p < 0.001, ns: not significant, Mann–Whitney U test, adjusted α-level = 0.013. ⊥ SEM, box and whisker plots indicate median, interquartile range (box), minimum and maximum (whiskers).

[Ca^2+^]_i_ of HTEpC assessed in Ca^2+^-free buffer solution remained constant under resting conditions (FURA-2 340/380 ratio: 1.00 ± 0.01). Application of 20:1 cafedrine/theodrenaline, cafedrine alone, and theodrenaline alone provoked a significant increase in [Ca^2+^]_i_ (FURA-2 340/380 ratio cafedrine/theodrenaline: 1.76 ± 0.06, cafedrine: 1.78 ± 0.24, theodrenaline: 2.68 ± 0.22; each *p* < 0.001; Fig. 3A,C,E). However, compared with Ca^2+^-containing buffer, peak [Ca^2+^]_i_ was significantly lower following the administration of cafedrine and theodrenaline alone (each *p* < 0.001, Fig. 3D,F), while peak [Ca^2+^]_i_ of 20:1 cafedrine/theodrenaline was comparable (*p* = 0.350, Fig. 3B). Interestingly, the theodrenaline-induced transient increase in [Ca^2+^]_i_ remained elevated and did not reach baseline during the rest of the observation period (Fig. 3E). β-adrenergic receptor stimulation leading to the transient increase in [Ca^2+^]_i_ was evaluated in subsequent experiments. Because the relevant influence of extracellular Ca^2+^ entry could not be ruled out following these experiments, all the subsequent experiments were performed in a Ca^2+^-free buffer solution.

### Adrenergic receptor stimulation is responsible for the rise in [Ca^2+^]_i_

**Figure 4:**
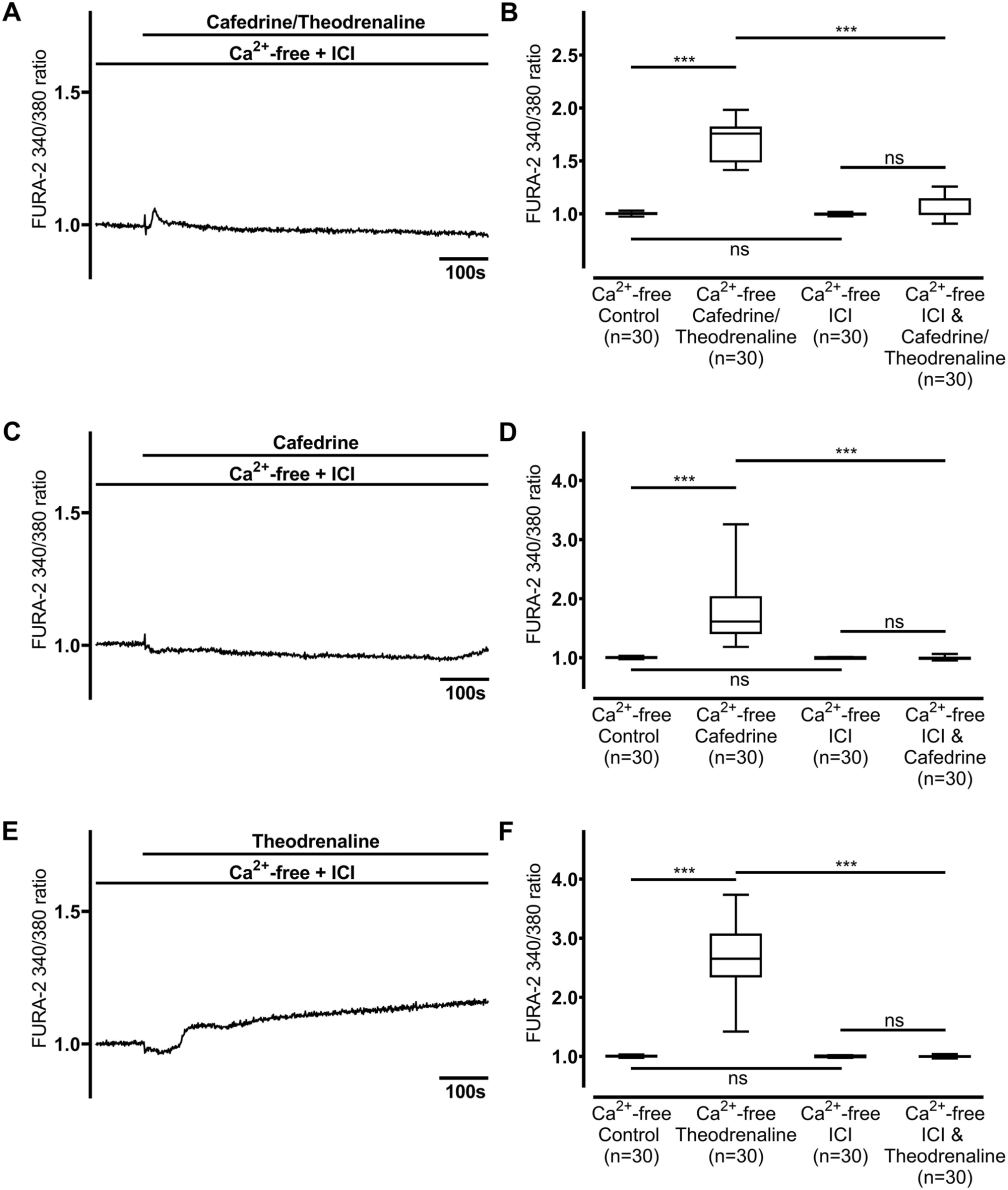
Ca^2+^ release is completely dependent on adrenergic receptor activation. When β-adrenergic receptors were blocked by high concentrations of the non-selective inhibitor ICI-118,551 (100 µM), no relevant peak [Ca^2^]_i_ was observed after the application of (**A, B**) 20:1 cafedrine/theodrenaline, (**C, D**) cafedrine alone, or (**E, F**) theodrenaline alone. The Ca^2+^-free control group is represented by the same 30 cells in each chart. FURA-2 340/380 ratio was normalized after a 100-s resting period, and each group consists of 30 cells from at least three different coverslips, ensuring independent measurements. Scale bar width represents 100 s. n = number of individual cells, ***p < 0.001, ns: not significant, Wilcoxon rank sum test was used to assess ICI-118,551 alone vs. drug preparation during inhibition, otherwise Mann–Whitney U test, adjusted α-level = 0.013. ⊥ SEM, box and whisker plots indicate median, interquartile range (box), minimum and maximum (whiskers).

When the non-selective blocker of β-adrenergic receptors, ICI-118,551 (100 µM), was applied, baseline [Ca^2+^]_i_ remained unaltered compared to the Ca^2+^-free controls (Fig. 4). However, 20:1 cafedrine/theodrenaline, cafedrine alone, and theodrenaline did not provoke any significant alteration in [Ca^2+^]_i_ in the presence of ICI-118,551 (cafedrine/theodrenaline: 1.05 ± 0.04, *p* = 0.416, Fig. 4A,B; cafedrine: 0.99 ± 0.01, *p* = 0.043 did not reach the adjusted α-level of 0.013, Fig. 4C,D; theodrenaline: 1.07 ± 0.10, *p* = 0.919, Fig. 4E,F). Because clinically relevant effects of other receptors or PDE could not be assumed from these data, further experiments elucidating these mechanisms were waived.

### Ca^2+^ is liberated from ER but not the mitochondria

**Figure 5:**
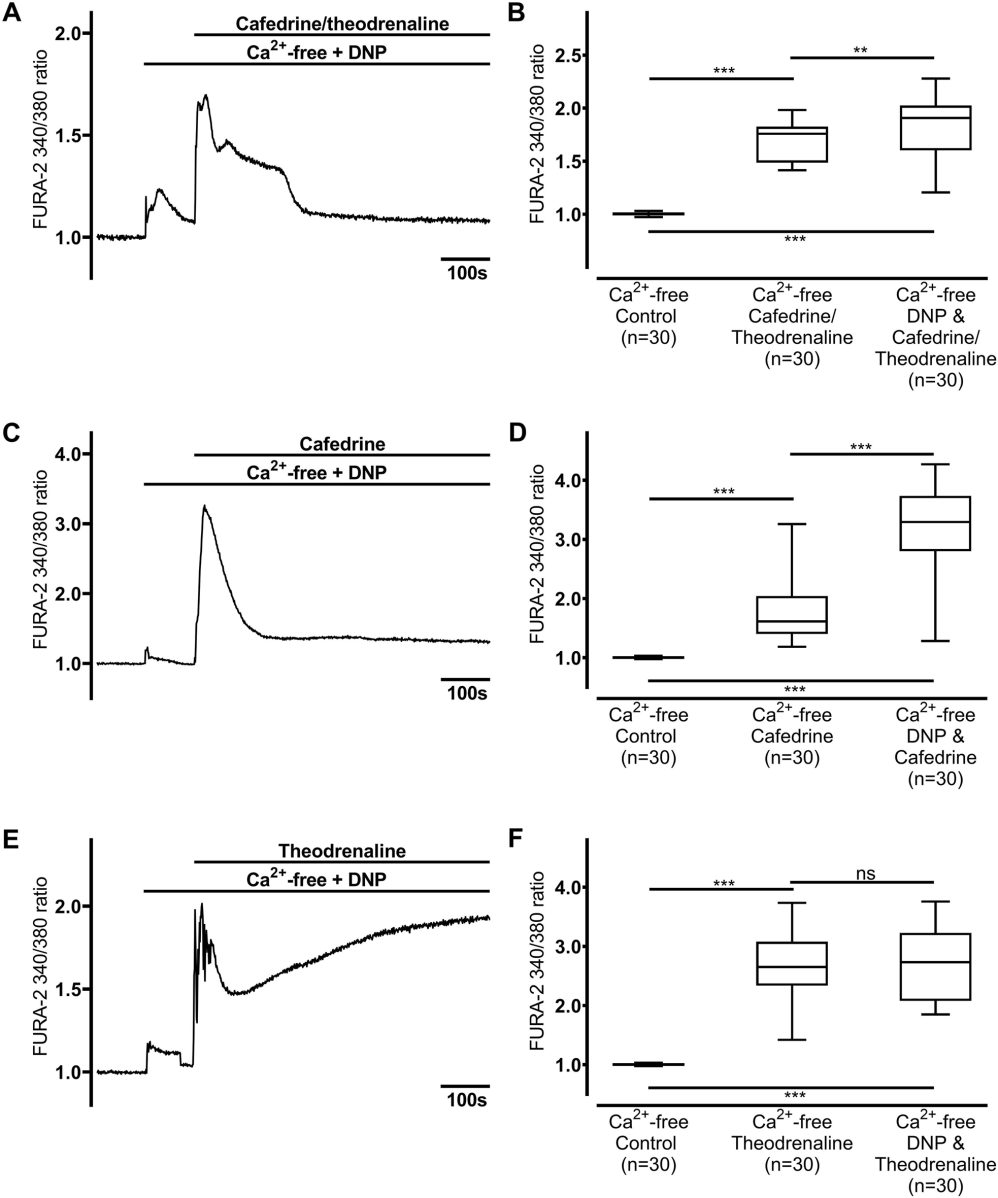
Ca^2+^ release occurs independently from mitochondrial Ca^2+^ stores. When mitochondrial stores were depleted by 2,4-dinitrophenol (DNP, 25 µM), [Ca^2+^]_i_ sharply increased following the application of (**A, B**) 20:1 cafedrine/theodrenaline, (**C, D**) cafedrine alone, and (**E, F**) theodrenaline alone. [Ca^2+^]_i_ did not return to its baseline value within the observation period. The Ca^2+^-free control group was represented by the same 30 cells in each chart. FURA-2 340/380 ratio was normalized after a 100-s resting period, and each group consists of 30 cells from at least three different coverslips, ensuring independent measurements. Scale bar width represents 100 s. n = number of individual cells, ***p < 0.001, ns: not significant, Mann–Whitney U test, adjusted α-level = 0.017. ⊥ SEM, box and whisker plots indicate median, interquartile range (box), minimum and maximum (whiskers).

**Figure 6:**
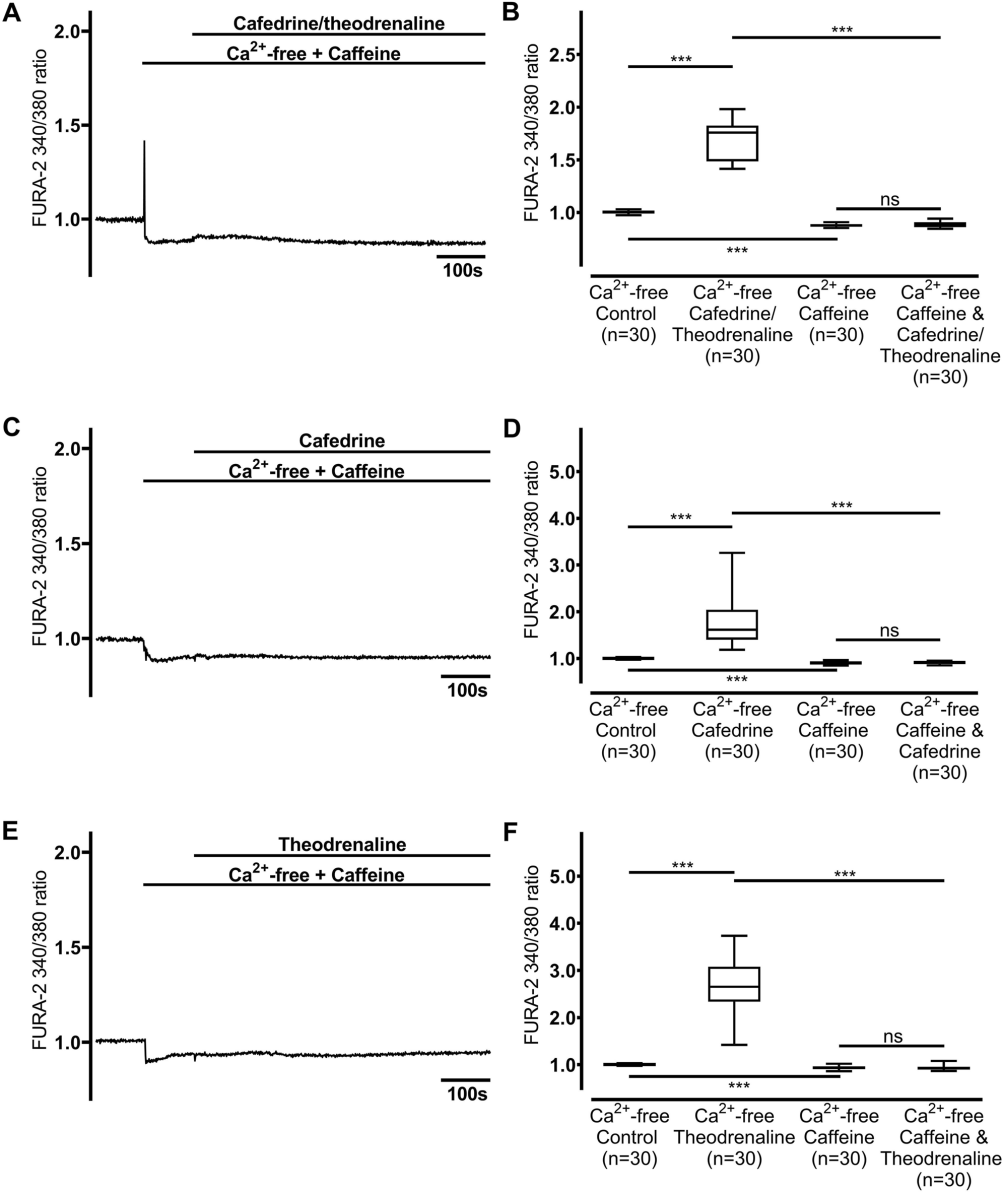
Ca^2+^ is released from intracellular caffeine-sensitive stores. When caffeine-sensitive stores were depleted by caffeine (30 mM), a subsequent decline in [Ca^2^]_i_ was observed, and no increase in [Ca^2^]_i_ was provoked by (**A, B**) 20:1 cafedrine/theodrenaline, (**C, D**) cafedrine alone, or (**E, F**) theodrenaline alone. The Ca^2+^-free control group was represented by the same 30 cells in each chart. FURA-2 340/380 ratio was normalized after a 100-s resting period, and each group consists of 30 cells from at least three different coverslips, ensuring independent measurements. Scale bar width represents 100 s. n = number of individual cells, ***p < 0.001, ns: not significant, Wilcoxon rank sum test was used to test caffeine alone vs. drug preparation during inhibition, otherwise Mann–Whitney U test, adjusted α-level = 0.013. ⊥ SEM, box and whisker plots indicate median, interquartile range (box), minimum and maximum (whiskers).

Because our experiments indicated intracellular Ca^2+^ release, further experiments were conducted to evaluate the origin of the intracellular Ca^2+^. First, mitochondrial Ca^2+^ stores were depleted using DNP (25 µM, Fig. 5). A small increase in [Ca^2+^]_i_ was observed following the administration of DNP, which quickly returned to its baseline; however, 20:1 cafedrine/theodrenaline, cafedrine alone, and theodrenaline alone still provoked a significant increase in [Ca^2+^]_i_ (cafedrine/theodrenaline: 1.17 ± 0.05, *p* < 0.001, Fig. 5A,B; cafedrine: 1.24 ± 0.16, *p* < 0.001, Fig. 5C,D; theodrenaline: 2.71 ± 0.27, *p* < 0.001, Fig. 5E,F). While peak values of [Ca^2+^]_i_ were comparable after the administration of theodrenaline (*p* = 0.994), 20:1 cafedrine/theodrenaline, and cafedrine alone, [Ca^2+^]_i_ peaks were significantly higher in the presence of DNP compared with the [Ca^2+^]_i_ peaks without depletion of mitochondrial Ca^2+^ stores (both *p* < 0.001). [Ca^2+^]_i_ did not reach baseline following the administration of cafedrine alone or theodrenaline alone. Furthermore, caffeine-sensitive Ca^2+^ stores, which are mainly represented by the ER, were depleted using caffeine (30 mM, Fig. 6). After caffeine was applied, a significant reduction in baseline [Ca^2+^]_i_ was observed, indicating Ca^2+^ store depletion (cafedrine/theodrenaline: 0.88 ± 0.01; cafedrine: 0.91 ± 0.01; theodrenaline: 0.90 ± 0.01; each *p* < 0.001). Subsequent application of 20:1 cafedrine/theodrenaline, cafedrine alone, or theodrenaline alone did not increase [Ca^2+^]_i_ (cafedrine/theodrenaline: 0.88 ± 0.01, *p* = 0.034 did not reach the adjusted α-level of 0.013, Fig. 6A,B; cafedrine: 0.91 ± 0.01, *p* = 0.114, Fig. 6C,D; theodrenaline: 0.93 ± 0.02; *p* = 0.299, Fig. 6E,F). Because the ER is a crucial caffeine-sensitive Ca^2+^ store, further experiments were conducted to evaluate the signal cascades triggering Ca^2+^ release from these stores.

### Increase in [Ca^2+^]_i_ is induced by RyR but not IP_3_ receptor activation

**Figure 7:**
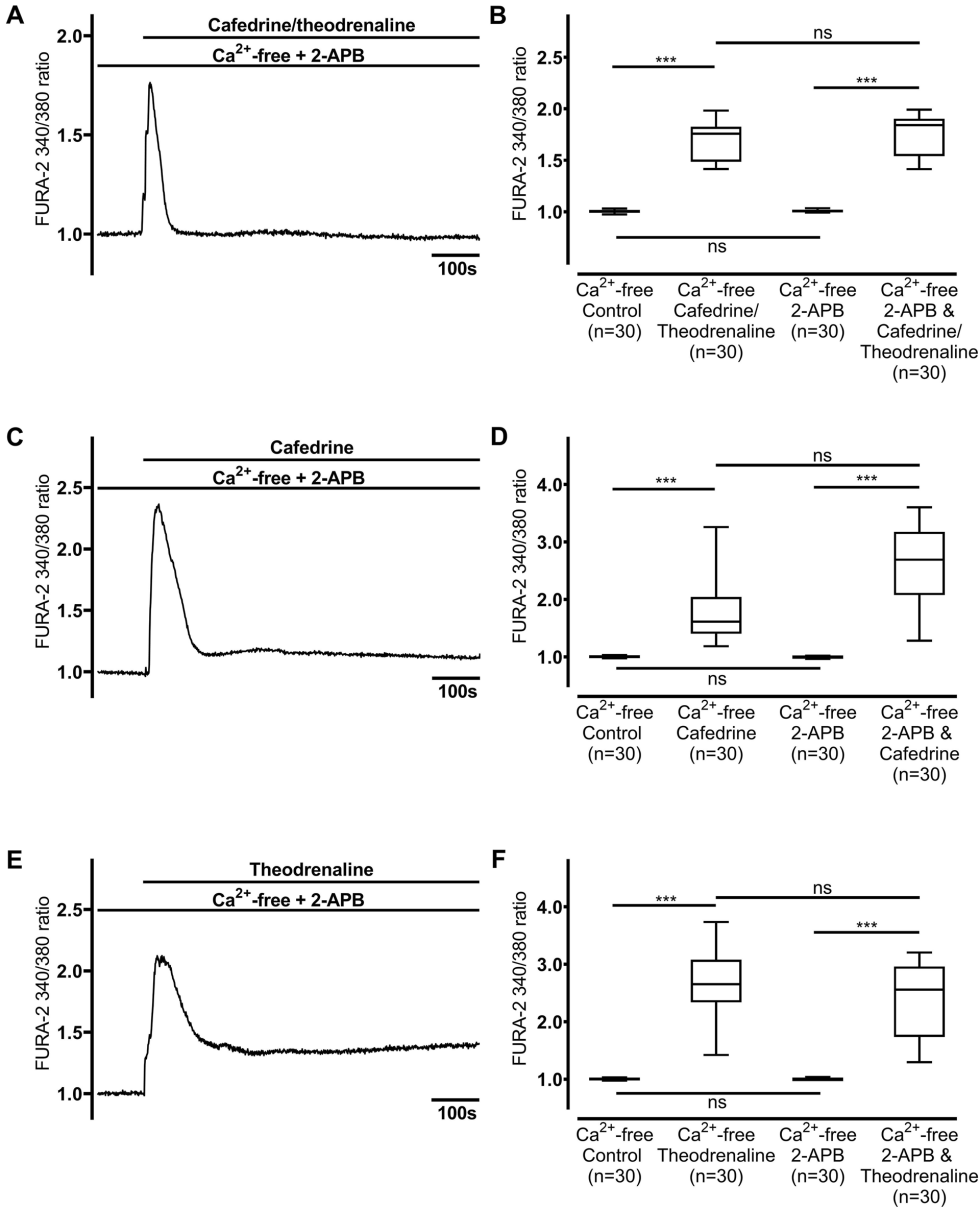
Ca^2+^ release from intracellular stores occurs independently from IP_3_ receptor activation. When IP_3_ receptors were inhibited using 2-APB (40 µM), maximum [Ca^2^]_i_ increased equally after the application of (**A, B**) 20:1 cafedrine/theodrenaline, (**C, D**) cafedrine alone, and (**E, F**) theodrenaline alone. The Ca^2+^-free control group was represented by the same 30 cells in each chart. FURA-2 340/380 ratio was normalized after a 100-s resting period, and each group consists of 30 cells from at least three different coverslips, ensuring independent measurements. Scale bar width represents 100 s. n = number of individual cells, ***p < 0.001, ns: not significant, Wilcoxon rank sum test was used to test 2-APB alone vs. drug preparation during inhibition, otherwise Mann–Whitney U test, adjusted α-level = 0.013. ⊥ SEM, box and whisker plots indicate median, interquartile range (box), minimum and maximum (whiskers).

**Figure 8:**
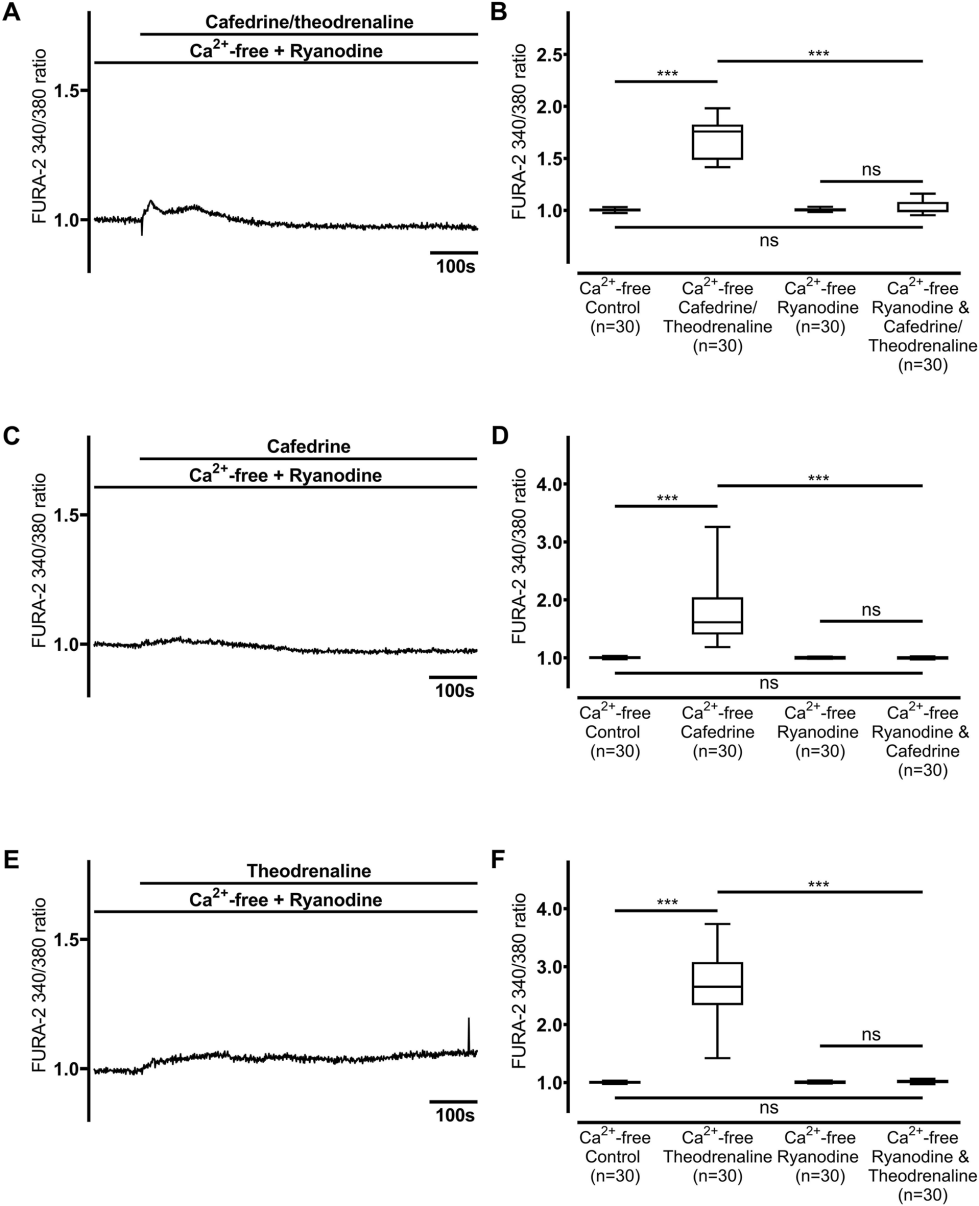
Ca^2+^ release from intracellular stores depends on ryanodine receptor activation. When ryanodine receptors were inhibited with ryanodine (40 µM), [Ca^2+^]_i_ remained unaltered after the application of (**A, B**) 20:1 cafedrine/theodrenaline, (**C, D**) cafedrine alone, and (**E, F**) theodrenaline alone. The Ca^2+^-free control group was represented by the same 30 cells in each chart. FURA-2 340/380 ratio was normalized after a 100-s resting period, and each group consists of 30 cells from at least three different coverslips, ensuring independent measurements. Scale bar width represents 100 s. n = number of individual cells, ***p < 0.001, ns: not significant, Wilcoxon rank sum test was used to test ryanodine alone vs. drug preparation during inhibition, otherwise Mann–Whitney U test, adjusted α-level = 0.013. ⊥ SEM, box and whisker plots indicate median, interquartile range (box), minimum and maximum (whiskers).

IP_3_ receptors were inhibited using 2-APB (40 µM, Fig. 7). While 2-APB did not alter baseline [Ca^2+^]_i_, application of 20:1 cafedrine/theodrenaline, cafedrine alone, or theodrenaline alone increased [Ca^2+^]_i_ to values at least equivalent to [Ca^2+^]_i_ without IP_3_ receptor inhibition (cafedrine/theodrenaline: 1.75 ± 0.08, *p* = 0.042 did not reach the adjusted α-level of 0.013, Fig. 7A,B; cafedrine: 2.58 ± 0.29, *p* < 0.001, Fig. 7C,D; theodrenaline: 2.39 ± 0.29, *p* = 0.173, Fig. 7E,F). In contrast, when RyR were inhibited using ryanodine (40 µM, Fig. 8), the increase in [Ca^2+^]_i_ following the application of cafedrine/theodrenaline, cafedrine alone, or theodrenaline alone vanished (cafedrine/theodrenaline: 1.02 ± 0.03, *p* = 0.416, Fig. 8A,B; cafedrine: 1.00 ± 0.01, *p* = 0.516, Fig. 8C,D; theodrenaline: 1.02 ± 0.01, *p* = 0.349, Fig. 8E,F). Because RyR activation was shown to be the crucial mechanism leading to Ca^2+^ release, RT-PCR was performed to identify the different RyR subtypes. Therefore, murine tracheae were used to ensure the availability of positive controls, ensuring accurate internal validity of the measurements.

### Expression of RyR in murine tracheae

*RyR-2* and *RyR-3* mRNA expression in both whole murine trachea and isolated tracheal epithelium (each n = 5) were identified by RT-PCR. *RyR-1* mRNA was not detected in the whole trachea or tracheal epithelium. An overview of the RT-PCR results, including the controls, is presented in Table 2, while Supplementary Fig. 1 illustrates the native PCR bands.

**Table 2:** RT-PCR identified ryanodine receptor (RyR)-2 and RyR-3 in murine whole trachea and tracheal epithelium.

	*Ryr1*	*Ryr2*	*Ryr3*
Skeletal muscle	+	−	+
Cardiac muscle	−	+	−
Diaphragm	+	−	+
Whole trachea	−	+	+
Tracheal epithelium	−	+	+
Negative control (H_2_O)	−	−	−

## Discussion

Our experiments revealed that 20:1 cafedrine/theodrenaline, cafedrine alone, or theodrenaline alone increased [Ca^2+^]_i_, when applied to human tracheal epithelial cells. We observed sharp, transient peaks in the FURA-2 340/380 ratio, which followed a dose–response relationship and were described by the Hill equation. Herein, it should be noted that the effect curves of 20:1 cafedrine/theodrenaline and cafedrine alone showed a profile where the increase from 0 to 100% is already achieved within one log unit, which would be classically attributed to ion channel activation. In contrast, only the dose–response curve of theodrenaline clearly indicated receptor-mediated effects because the increase from 0 to 100% required two log units. However, although the formal criteria for receptor-mediated Ca^2+^ release were only barely met in our dose–response curves following the application of 20:1 cafedrine/theodrenaline and cafedrine alone, our following experiments clearly proved adrenergic and ryanodine receptor-mediated Ca^2+^ release following the application of all three substances analyzed in our experiments. Interestingly, the EC_50_ of cafedrine alone and theodrenaline alone were within comparable ranges; however, when applied as the clinically used 20:1 mixture, much more cafedrine was required to achieve a significant effect on [Ca^2+^]_i_, and the applied concentration of theodrenaline seemed almost negligible in the light of the calculated EC_50_ of theodrenaline alone. Moreover, [Ca^2+^]_i_ peaks observed after the application of 20:1 cafedrine/theodrenaline were consistently lower than those observed following applications of the individual substances alone. Interestingly, clinically irrelevant high concentrations of 20:1 cafedrine/theodrenaline ultimately led to cell lysis, which was also observed following the administration of high concentrations of cafedrine or theodrenaline alone; however we never observed a higher FURA-2 340/380 ratio than that shown in our dose–response curves. Although the pharmacokinetics of cafedrine and theodrenaline remain largely unknown, the immediate response in blood pressure was attributed to theodrenaline, while the effects of cafedrine were observed after a 20-min delay^17,20^. Therefore, it has been hypothesized that cafedrine may not provoke any sympathomimetic actions alone; it may be metabolized into active metabolites that exert its clinical effects^17^. In our experiments, the effect observed after the application of 20:1 cafedrine/theodrenaline could only be attributed to theodrenaline alone if strong synergistic effects would apply in the presence of cafedrine. Because the concentration of theodrenaline alone in the 20:1 mixture was insufficient to provoke any significant changes in [Ca^2+^]_i_, we conclude that the foremost effect on changing [Ca^2+^]_i_ in our experiments was induced by cafedrine. This assumption is supported by the observation that very high concentrations of cafedrine were applied using the 20:1 combination compared with the dose–response relationship of cafedrine alone. However, our model cannot represent the effects of potential drug metabolites, as we used isolated cells, and the drugs were directly applied to the buffer solution. Furthermore, clinical data reported a plasma concentration of 6 µg/ml after the intravenous application of one ampoule of 2 ml cafedrine/theodrenaline; therefore, all concentrations used in our experiments were significantly higher than clinically used concentrations^20^. However, the transient intravascular and intraepithelial concentrations immediately after injection are unknown and might be much higher than those observed after distribution to the body compartments; therefore, our data should be interpreted with caution, and further studies are necessary to evaluate the transferability of the concentrations used in our experiments.

After the transient peak, during which Ca^2+^ directly increases ciliary beat frequency and calmodulin-bound Ca^2+^ activated cyclic guanosine monophosphate (cGMP) and cyclic adenosine monophosphate (cAMP)-dependent pathways, [Ca^2+^]_i_ rapidly returned to its baseline^3^. The rapid restoration of baseline [Ca^2+^]_i_ is primarily explained by SERCA activity, which pumps Ca^2+^ into the ER after cytosolic Ca^2+^ release, or by mitochondrial Ca^2+^ buffering^21,22^. However, almost every other small cell organelle contributes to the rapid restoration of baseline [Ca^2+^]_i_, and prolonged alteration of ciliary beat frequency is induced by transient changes in [Ca^2+^]_i_. Immediately after purinergic or cholinergic stimulation, tracheal epithelial cells exhibited a transient increase in [Ca^2+^]_i_; however, ciliary beat frequency remained consistently increased^23,24^. Therefore, we conclude that analogous mechanisms are mediated after the stimulation of β-adrenergic receptors, which were the pivotal receptors involved in [Ca^2+^]_i_ alterations in our experiments. The increase in [Ca^2+^]_i_ completely vanished after 20:1 cafedrine/theodrenaline, cafedrine alone, or theodrenaline alone were added during non-selective adrenergic receptor inhibition. These results are in line with the clinical effect of cafedrine/theodrenaline, which increases cardiac stroke volume via β_1_-adrenergic receptor activation^14^. In-vitro studies using human atrial myocardium and coronary arteries also elucidated β_1_-adrenergic receptor activation as a pivotal mechanism; however, effects on α-adrenergic receptors were observed in arteries after β-adrenergic receptor inhibition^17,25^. Although these effects were attributed to theodrenaline alone, we did not observe similar effects in human tracheal epithelial cells. These observations are in line with the data reported by Weiterer et al., who reported the exclusive presence of the α1D-adrenergic receptor subtype in murine tracheal epithelium^26^. However, murine particle transport velocity was independent from α-adrenergic receptor activation. Therefore, we conclude that α-receptor activation might occur in human tracheal epithelial cells following the administration of cafedrine/theodrenaline, but no influence on [Ca^2+^]_i_ or mucociliary clearance could be detected in light of the available in-vitro data^26^. The theophylline component of cafedrine and theodrenaline is believed to inhibit PDE, which should lead to the persistence of second messengers, such as cGMP and cAMP, improving cardiac inotropy^14^. However, only high, clinically irrelevant concentrations of cafedrine/theodrenaline were able to provoke significant inhibition of PDE in human atrial myocardium^17^. Although we used high concentrations of cafedrine/theodrenaline, cafedrine alone, and theodrenaline alone, we were only able to detect β-adrenergic receptor stimulation because the increase in [Ca^2+^]_i_ completely vanished in the presence of β-adrenergic receptor inhibition. When other signal transduction cascades were involved, we should have detected a persistent increase in [Ca^2+^]_i_. This finding is underlined by the knowledge that PDE inhibition might not influence ciliary beat frequency or mucociliary clearance to a clinically relevant degree, because data remain controversial regarding the alteration of mucociliary clearance following treatment with theophylline^27–29^.

When we used Ca^2+^-free buffer solution, [Ca^2+^]_i_ increased to a significantly lesser degree following administration of cafedrine alone and theodrenaline alone than the increase observed in Ca^2+^-containing buffer solution. Therefore, extracellular Ca^2+^ influx contributes to the rise in [Ca^2+^]_i_, which is foremost realized through SOCE in non-excitable cells following Ca^2+^ release from internal stores^12,13^. ORAI proteins, which are mediated by stromal interaction molecule proteins, are the most important mediators of SOCE^12^. Therefore, further experiments elucidating receptor expression and distinct signal transduction pathways should be performed, although their specific inhibition is complicated due to their diverse interactions and multiple targets located on the plasma membrane^13^. However, because the [Ca^2+^]_i_ peak in the Ca^2+^-free buffer solution was comparable to the peak observed in Ca^2+^-containing buffer solution following the administration of 20:1 cafedrine/theodrenaline, the clinical relevance of SOCE following the administration of cafedrine/theodrenaline in human tracheal epithelial cells remains questionable. Internal stores were depleted to detect the intracellular stores, which released Ca^2+^ following the administration of cafedrine/theodrenaline. When mitochondrial Ca^2+^ stores were depleted, [Ca^2+^]_i_ continued to increase following the administration of 20:1 cafedrine/theodrenaline, cafedrine alone, or theodrenaline alone. Therefore, we conclude that cafedrine/theodrenaline does not depolarize mitochondrial membrane potential, which would lead to Ca^2+^ release from these stores. However, [Ca^2+^]_i_ peaks following the administration of 20:1 cafedrine/theodrenaline and cafedrine alone were significantly higher than those observed without prior mitochondrial store depletion. This observation confirms the mitochondrial ability to buffer Ca^2+^ ions via the mitochondrial Ca^2+^ uniporter when [Ca^2+^]_i_ exceeds 500 nM^30^. Interestingly, [Ca^2+^]_i_ remained higher for the rest of the observation time. Because DNP administration decouples oxidative phosphorylation without altering cytosolic pH, less ATP supplying the ATP-dependent SERCA might be available in these experiments, leading to the observation of persistent [Ca^2+^]_i_ baseline shift. However, SERCA was still able to handle the transient peak [Ca^2+^]_i_ by altering [Ca^2+^]_i_ close to its former baseline value. This finding underlines our conclusion that the elevated peak in [Ca^2+^]_i_ is foremost triggered by the inhibited mitochondrial Ca^2+^ capacity and not by a lack of ATP, which might impair SERCA activity.

Because the increase in [Ca^2+^]_i_ completely vanished following the administration of 20:1 cafedrine/theodrenaline, cafedrine alone, or theodrenaline alone, when RyR were inhibited, we conclude that cafedrine/theodrenaline releases Ca^2+^ from the ER exclusively by RyR activation. In general, Ca^2+^ release from the ER is achieved via IP_3_ receptor or RyR activation^31^. However, our experiments revealed equal peaks of [Ca^2+^]_i_ when IP_3_ receptors were inhibited, and the increase in [Ca^2+^]_i_ completely vanished after RyR inhibition. Therefore, we conclude that Ca^2+^ release following β-adrenergic receptor activation depends solely on RyR activation, and IP_3_ receptor-associated Ca^2+^ release does not occur in human tracheal epithelial cells following cafedrine/theodrenaline administration.

Consequently, RyR-2 and RyR-3 expression was revealed using RT-PCR in murine tracheal epithelium. While RyR-3 is co-expressed with RyR-1 or RyR-2 in many tissues, RyR 2 has mainly been studied in cardiac muscle cells; however, RyR-2 expression has been demonstrated in smooth muscle cells and non-excitable cells, such as pancreatic acinar cells and kidney epithelial cells^32–35^. Therefore, RyR-2 expression in the tracheal epithelium is in line with its expression in other non-excitable cells. While RyR activation is achieved following various signal transduction cascades, the best-known mechanism is the Ca^2+^-induced Ca^2+^-release, whereby RyR-2 activation is triggered by a local increase of [Ca^2+^]_i_^36^. Local increase in [Ca^2+^]_i_ can be realized through nearby RyR activation, or IP_3_ receptor activation; however, our data indicate that RyR activation was independent from IP_3_ receptor activation following cafedrine/theodrenaline administration^37^. Therefore, alternative RyR activation following β-adrenergic receptor stimulation must be considered in tracheal epithelial cells. β-adrenergic stimulation has been shown to increase RyR-2 activity in cardiac muscle cells via intracellular-mediated Ca^2+^ and Mg^2+^ regulation, and receptor phosphorylation^38^. Furthermore, protein kinase A and cAMP, which are both pivotal messengers following the β_1_-signal transduction cascade, have been shown to induce Ca^2+^ release via RyR-2 in cardiac muscles and non-excitable cells, respectively^39,40^. In addition, adrenergic receptor signaling increases nicotinic acid adenine dinucleotide phosphate and cyclic adenosine diphosphate-ribose levels, which both activate RyR-2-associated Ca^2+^ release^33,41^. Because RyR-3 is more readily activated by an increase in local [Ca^2+^]_i_ compared with RyR-2, its activation in tracheal epithelial cells following the administration of cafedrine/theodrenaline can be achieved following RyR 2-associated Ca^2+^ efflux^42^. However, as discrepancies in receptor expression between mammals could not be excluded, further studies in human tissues should be conducted to confirm RyR expression. Local distribution, and the RyR-2-to-RyR-3 ratio in tracheal epithelial cells could be evaluated using immunohistochemistry.

Several limitations of our experiments must be acknowledged. First, we used isolated tracheal epithelial cells; therefore, the physiological integrity of a respiratory tract was not preserved, including a lack of basal tissues and cell–cell junctions. Therefore, physiological drug administration via capillary vessels could not be replicated, and atypical entrance (e.g. from the apical or lateral side of the cells) of our tested drugs was preserved. Second, we used high concentrations of cafedrine/theodrenaline, cafedrine, and theodrenaline to achieve alterations in [Ca^2+^]_i_ and clinically administered concentrations are much lower; therefore, it is possible that we observed [Ca^2+^]_i_ kinetics that do not occur when lower concentrations are used in vivo. Third, some concentrations applied to achieve the specific dose–response curves were higher than was warranted by the integrity of the observed cells; therefore, a maximum FURA-2 340/380 ratio following substance administration was set at the last valid observed value. However, concentrations applied to assess the distinct signal transduction cascades did prove the validity of the calculated dose–response curves in our experimental setting. Fourth, our measurement of [Ca^2+^]_i_ using the FURA-2 340/380 ratio was not calibrated; therefore, we can only report relative alterations in [Ca^2+^]_i_, and no absolute concentrations were measured.

In conclusion, we provide evidence that cafedrine/theodrenaline, cafedrine alone, or theodrenaline alone induce the release of Ca^2+^ from caffeine-sensitive internal stores that is exclusively triggered by β-adrenergic receptor stimulation, resulting in RyR activation. RT-PCR revealed the presence of RyR-2 and RyR-3 in mammalian cells, and the relevant influence of extracellular Ca^2+^ influx was only observed after the application of cafedrine alone or theodrenaline alone. However, clinical plasma concentrations are considerably lower than those used in our experiments to trigger a significant increase in [Ca^2+^]_i_; therefore, further studies are needed to alter the ability of cafedrine/theodrenaline to change mucociliary clearance in clinical practice.

### Supplementary Information

Supplementary Figure 1.

## Data Availability

The datasets used and/or analysed during the current study are available from the corresponding author on reasonable request.

## Abbreviations

2-APB: 2-Aminoethoxydiphenylborane
[Ca^2+^]_i_: Intracellular Ca^2+^ concentration
cAMP: Cyclic adenosine monophosphate
cGMP: Cyclic guanosine monophosphate
DMSO: Dimethyl sulfoxide
DNP: 2,4-Dinitrophenol
EC_50_: Median effective concentration
ER: Endoplasmic reticulum
HEPES: 4-(2-Hydroxyethyl)-1-piperazineethanesulfonic acid
IP_3_: Inositol trisphosphate
KCl: Potassium chloride
mRNA: Messenger ribonucleic acid
PDE: Phosphodiesterases
RT-PCR: Reverse transcription polymerase chain reaction
RyR: Ryanodine receptor
SERCA: Sarcoplasmic/endoplasmic reticulum calcium ATPase
SOCE: Store-operated Ca^2+^ entry
