# Supplementary material for: A fixed 20:1 combination of cafedrine/theodrenaline increases cytosolic Ca2+ concentration in human tracheal epithelial cells via ryanodine receptor-mediated Ca2+ release
Source: Sci Rep. 2023 Sep 27;13:16216. doi: 10.1038/s41598-023-43342-0 (PMC10533847; doi:10.1038/s41598-023-43342-0)
Supplement: Supplementary file 1 — Supplementary Figure 1. [file 41598_2023_43342_MOESM1_ESM.pdf]

A

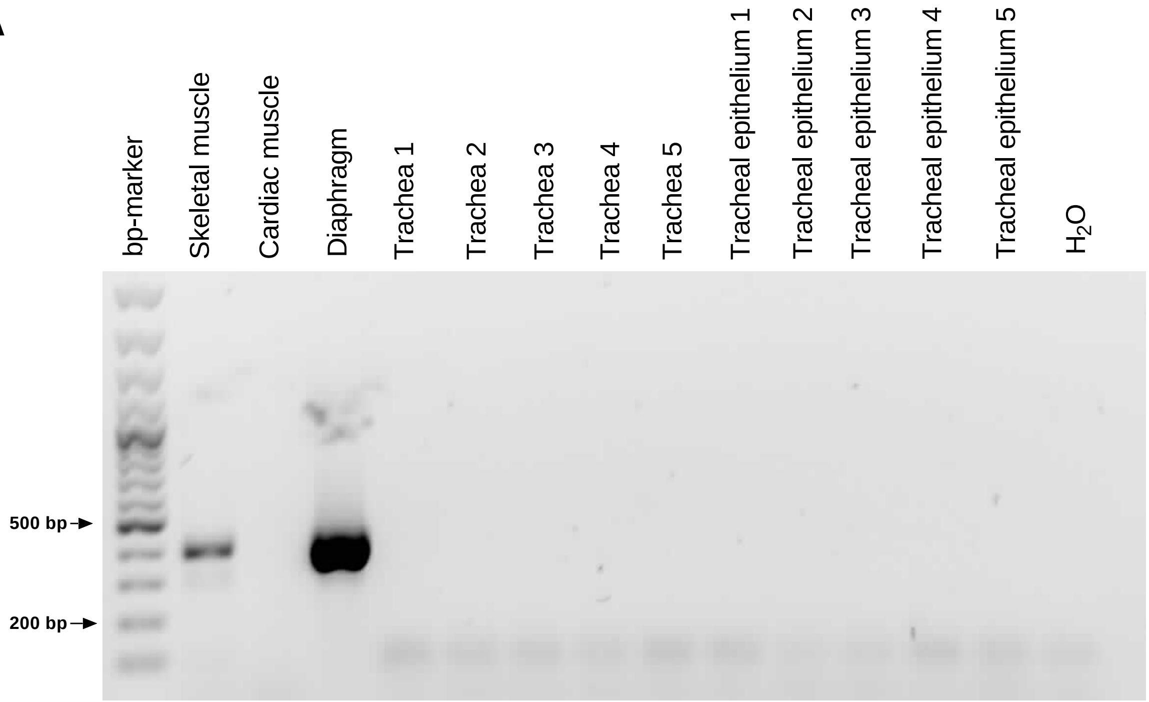
bp-marker
Skeletal muscle
Cardiac muscle
Diaphragm
Trachea 1
Trachea 2
Trachea 3
Trachea 4
Trachea 5
Tracheal epithelium 1
Tracheal epithelium 2
Tracheal epithelium 3
Tracheal epithelium 4
Tracheal epithelium 5
H_2O
500 bp
200 bp

B

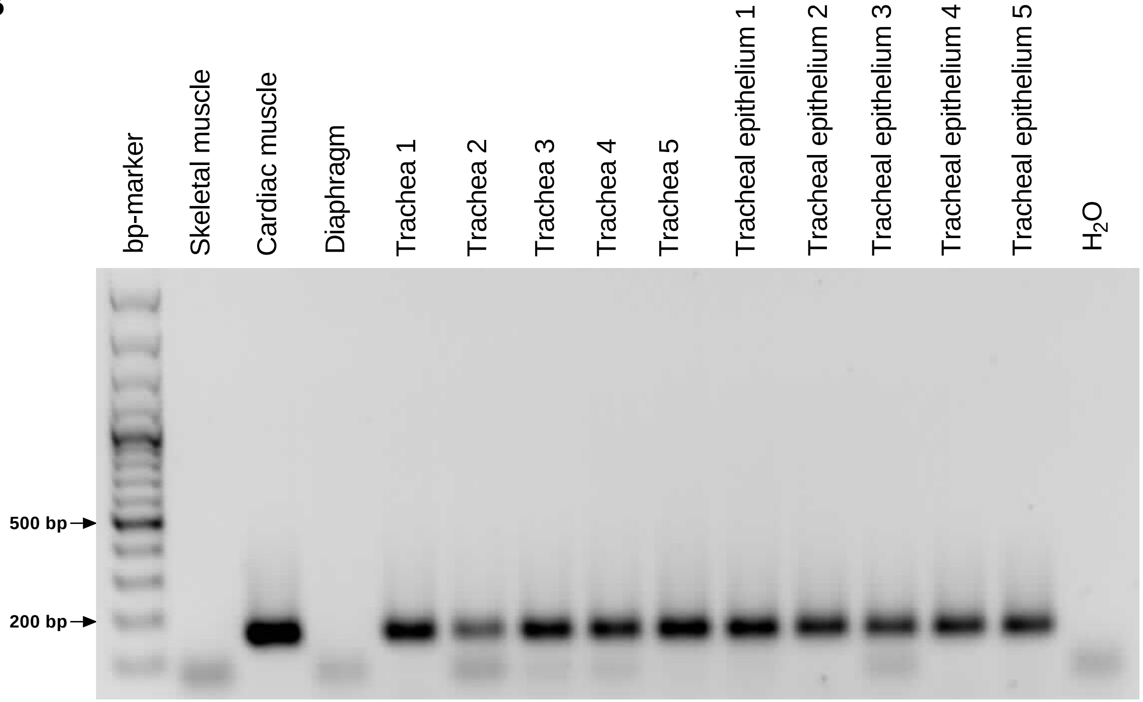
bp-marker
Skeletal muscle
Cardiac muscle
Diaphragm
Trachea 1
Trachea 2
Trachea 3
Trachea 4
Trachea 5
Tracheal epithelium 1
Tracheal epithelium 2
Tracheal epithelium 3
Tracheal epithelium 4
Tracheal epithelium 5
H_2O
500 bp
200 bp

C

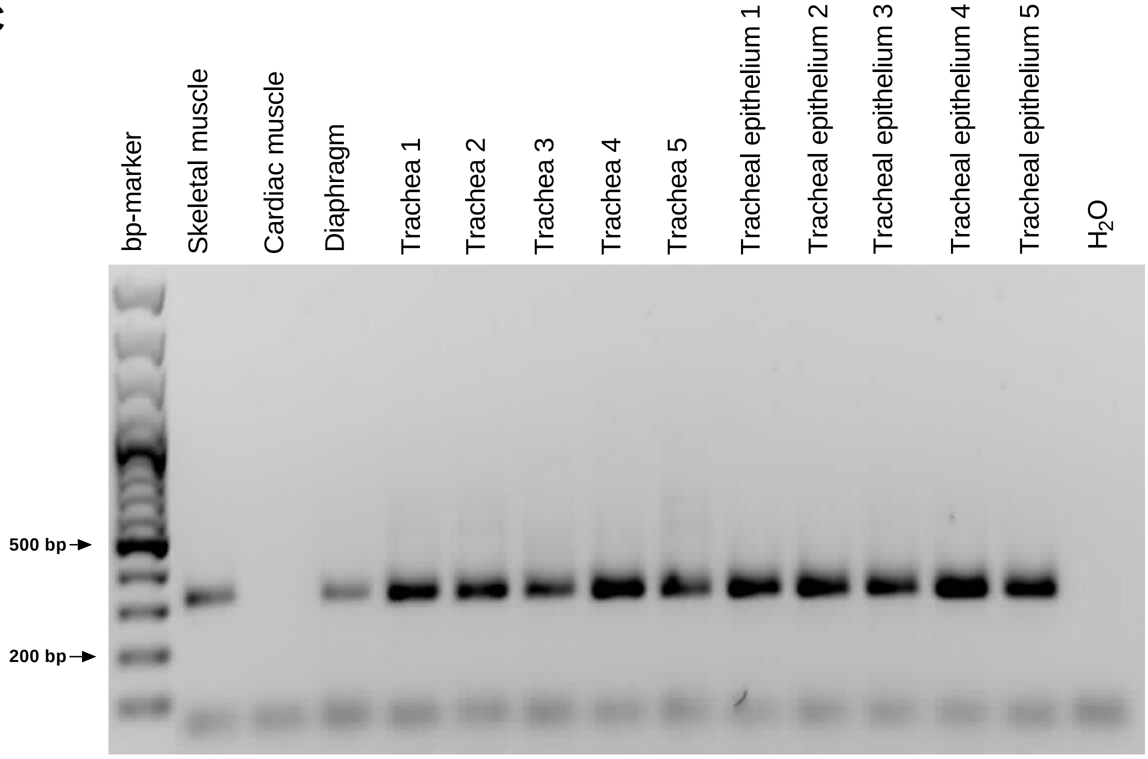

Supplementary Figure 1. Expression analysis in mouse tissues using reverse transcriptase (RT)-PCR. **A** Ryanodine receptor (RyR)-1 (*Ryr*1) was not detected in murine trachea or tracheal epithelium, whereas transcripts encoding **B** RyR-2 (*Ryr*2) and **C** RyR-3 (*Ryr*3) were detected.
